# Calcium polystyrene sulfonate-induced rectal ulcer causing *E. coli* native-valve infective endocarditis

**DOI:** 10.1007/s12328-024-01949-4

**Published:** 2024-03-25

**Authors:** Shinnosuke Fukushima, Hideharu Hagiya, Hiroyuki Honda, Tomoharu Ishida, Ryohei Shoji, Kou Hasegawa, Fumio Otsuka

**Affiliations:** 1 Department of General Medicine, Okayama University Graduate School of Medicine, Dentistry and Pharmaceutical Sciences, 2-5-1 Shikata-Cho, Kitaku, Okayama 700-8558 Japan; 2 Department of Gastroenterological Surgery, Okayama University Graduate School of Medicine, Dentistry and Pharmaceutical Sciences, Okayama, 700-8558 Japan

**Keywords:** Bacteremia, Calcium polystyrene sulfonate, *Escherichia coli*, Infective endocarditis, Rectal ulcer

## Abstract

*Escherichia coli*-associated native-valve infective endocarditis is a rare disease that affects elderly patients with underlying risk factors such as diabetes mellitus, malignancy, and renal failure. Long-term use of calcium polystyrene sulfonate is a potential risk factor for gastrointestinal mucosal damage or even colorectal ulcers. Herein, we describe a fatal case of a 66-year-old Japanese man with diabetes mellitus and renal failure who was prescribed calcium polystyrene sulfonate (CPS) for 11 years and developed a CPS-induced rectal ulcer, leading to *E. coli* native-valve infective endocarditis. The patient was admitted to our hospital due to acute-onset impaired consciousness. As a result of the systemic investigation, he was diagnosed with *E. coli* bacteremia accompanied by multiple cerebral infarctions and an acute hemorrhagic rectal ulcer. Transesophageal echocardiography revealed a 20-mm vegetative structure on the mitral valve, resulting in a final diagnosis of *E. coli*-associated infective endocarditis. After rectal resection, mitral valve replacement surgery was performed; however, the patient died shortly after surgery. Pathological findings of the resected rectum showed deposition of a basophilic crystalline material suggesting the presence of CPS. Our case highlights the potential risk of colorectal ulcers in a long-term CPS user, which can trigger bacterial translocation and endocarditis as fatal complications.

## Introduction

*Escherichia coli* bacteremia is the most common cause of community-onset bloodstream infections, with frequent sources from the urinary or biliary tract [1, 2]; however, clinical cases of infective endocarditis (IE) are rare [3]. *E. coli*-associated IE was reported to account for approximately 0.5–1.8% of IE cases, with a mortality rate of 17–24% and cardiac surgery rate of 42–51% [3–5]. *E. coli* potentially invades the native valves or myocardium without underlying prosthetic valves in elderly patients with risk factors, including diabetes, malignancy, excessive alcohol consumption, and renal failure [3–6]. According to a literature review, 52% of *E. coli*-associated IE was preceded by urinary tract infection, while an infectious source was unclear in 48% of the cases [3]. Although a few such cases originating from biliary tract infections were reported [7, 8], no documentation possibly exists regarding *E. coli*-associated IE precipitated by intestinal lesions.

Calcium polystyrene sulfonate (CPS) is a cation-exchange resin that is frequently used in patients with hyperkalemia. Well-known adverse effects of the drug include hard stools and constipation, potentially resulting in colon ulcers and intestinal perforation [9, 10]. Based on previous cases, intramucosal deposition of CPS is assumed to directly induce intestinal mucosal damage [10, 11]. Cardiovascular risks, such as coronary heart disease, type 2 diabetes mellitus, and chronic kidney disease, are supposedly relevant factors for CPS-associated intestinal mucosal damage [9]. Gastrointestinal complications induced by the crystals of ion-exchange resins and CPS, such as inflammation, necrosis, and dysfunction of the digestive mucous membrane, have been clinically reported to date [11]. However, to the best of our knowledge, IE cases preceded by CPS-induced intestinal damage are yet to be clearly described.

Herein, we report a case of *E. coli*-associated native-valve IE, potentially developed after CPS-induced rectal ulcers, which involved an older patient with diabetes mellitus and renal failure undergoing hemodialysis.

## Case report

A 66-year-old Japanese male patient who lived an independent daily life was hospitalized at a hospital 1 month ago for postoperative management of a lower extremity fracture due to a fall. He developed a sudden-onset unconsciousness and was transferred to our hospital for systemic investigation. His medical history included type 2 diabetes mellitus, hemodialysis for > 20 years, and mild aortic and mitral valve regurgitation. His medications included CPS, clonazepam, lansoprazole, nalfurafine, ferric citrate hydrate, lanthanum carbonate hydrate, pregabalin, polaprezinc, and loxoprofen sodium hydrate. Of these, CPS had been administered for 11 years for the treatment of hyperkalemia due to renal failure.

**Fig. 1:**
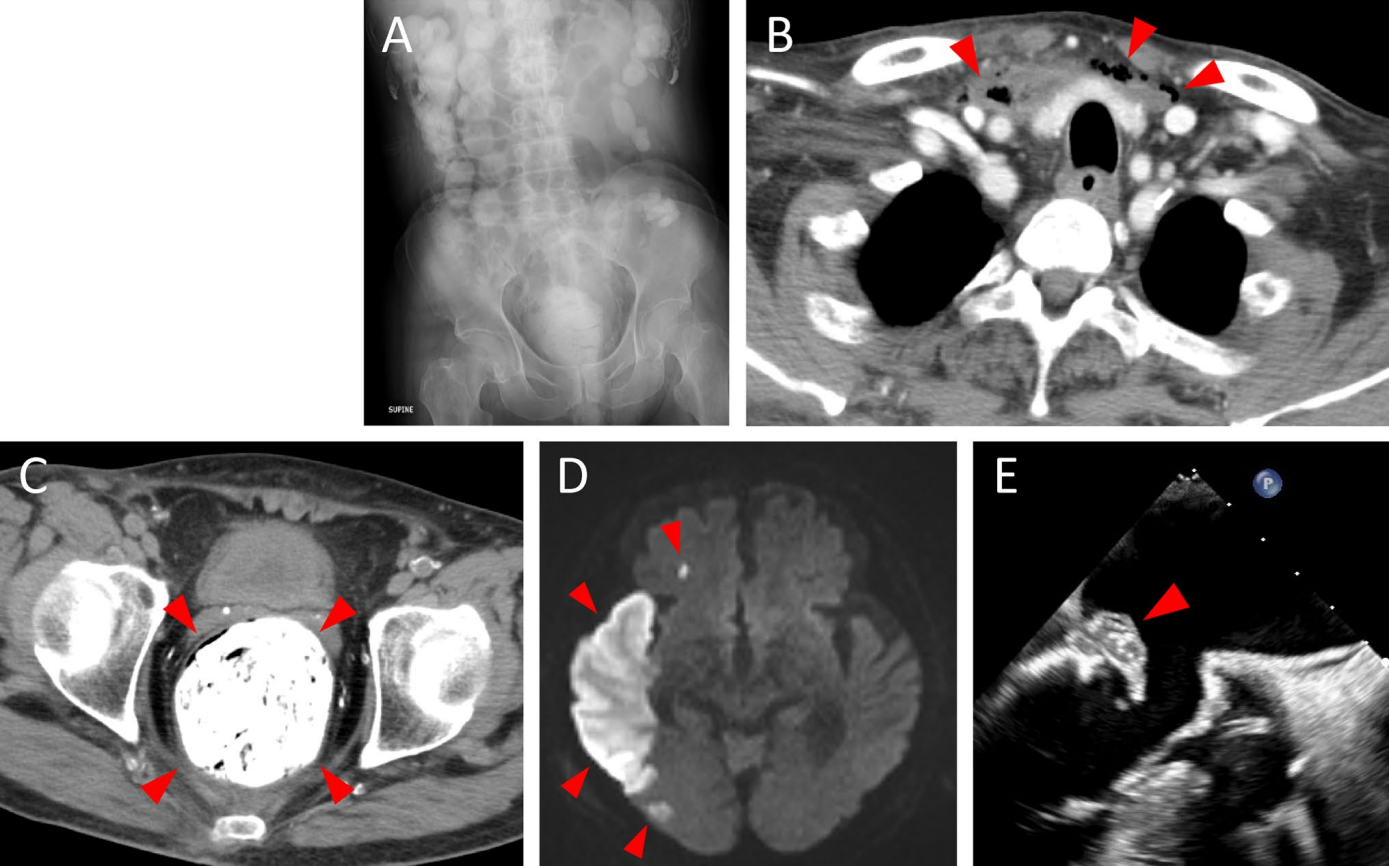
Radiological and ultrasound findings of the patient. **A** Abdominal radiography showing severe constipation. **B** Chest computed tomography showing gas production along the anterior mediastinum (arrowheads). **C** Abdominal computed tomography showing hard stools at the rectal colon (arrowheads). **D** Head diffusion-weighted magnetic resonance imaging showing multiple cerebral infarctions (arrowheads). **E** Transesophageal echocardiography showing a 20-mm-sized vegetative structure on the damaged mitral valve (arrowheads)

On admission, he was drowsy, and his vital signs were as follows: blood pressure, 158/69 mmHg; heart rate, 85 beats per min; respiratory rate, 24 per min; saturation of percutaneous oxygen, 100% on room air; and body temperature, 37.2 °C. Laboratory tests showed elevations of C-reactive protein (23.2 mg/dL), hemoglobin A_1_C (8.2%), d-dimer (22.2 μg/mL), and reduced platelet count (13,000 /μL) (Table 1). Abdominal radiography showed severe constipation (Fig. 1A), and computed tomography revealed gas production along the anterior mediastinum and hard stool in the rectal colon (Fig. 1B, C). Magnetic resonance imaging of the head revealed multiple cerebral infarctions without aneurysms, and the right middle cerebral artery was poorly visualized (Fig. 1D). Transthoracic echocardiography did not reveal any findings suggesting endocarditis at that time. An empiric therapy with vancomycin and meropenem was initiated under the tentative diagnosis of sepsis and disseminated intravascular coagulation. Blood cultures drawn on admission and on the 5th day of admission tested positive for *E. coli*. A urine culture was not obtained because the patient was on dialysis and anuric. The infectious focus of *E. coli* bacteremia could not be identified either in the post-surgical site of bone fracture or the biliary tract. Due to a persistent state of unconsciousness, the patient was bedridden.

**Table 1:** Laboratory data on admission

WBC	7560	/μL	D-bil	1.6	mg/dL	Ca	8.1	mg/dL
RBC	4.3 × 10^6^	/μL	LDH	212	U/L	IP	2.0	mg/dL
Hb	12.8	g/dL	CK	589	U/L	PT	12.2	s
PLT	1.3 × 10^4^	/μL	AMY	22	U/L	PT (%)	74	%
TP	4.4	g/dL	UN	62.4	mg/dL	PT-INR	1.2	
Alb	1.9	g/dL	Cr	7.3	mg/dL	APTT	44.2	s
AST	45	U/L	CRP	23.2	mg/dL	Fibrinogen	390	mg/dL
ALT	17	U/L	Na	140	mEq/L	FDP	31.6	ng/mL
Γ-GT	77	U/L	K	3.5	mEq/L	D-dimer	22.2	μg/mL
T-bil	2.3	mg/dL	Cl	102	mEq/L	AT III	34	%

*WBC* white blood cell, *RBC* red blood cell, *Hb* hemoglobin, *PLT* platelet, *TP* total protein, *Alb* albumin, *AST* aspartate aminotransferase, *ALT* alanine aminotransferase, *Γ-GT* Γ-glutamyl transpeptidase, *T-bil* total bilirubin, *D-bil* direct bilirubin, *LDH* lactate dehydrogenase, *CK* creatine kinase, *AMY* amylase, *UN* urea nitrogen, *Cr* creatinine, *CRP* C-reactive protein, *IP* inorganic phosphorus, *PT* prothrombin time, *APTT* activated partial thromboplastin time, *FDP* fibrinogen/fibrin degradation products, *AT III* antithrombin III

**Fig. 2:**
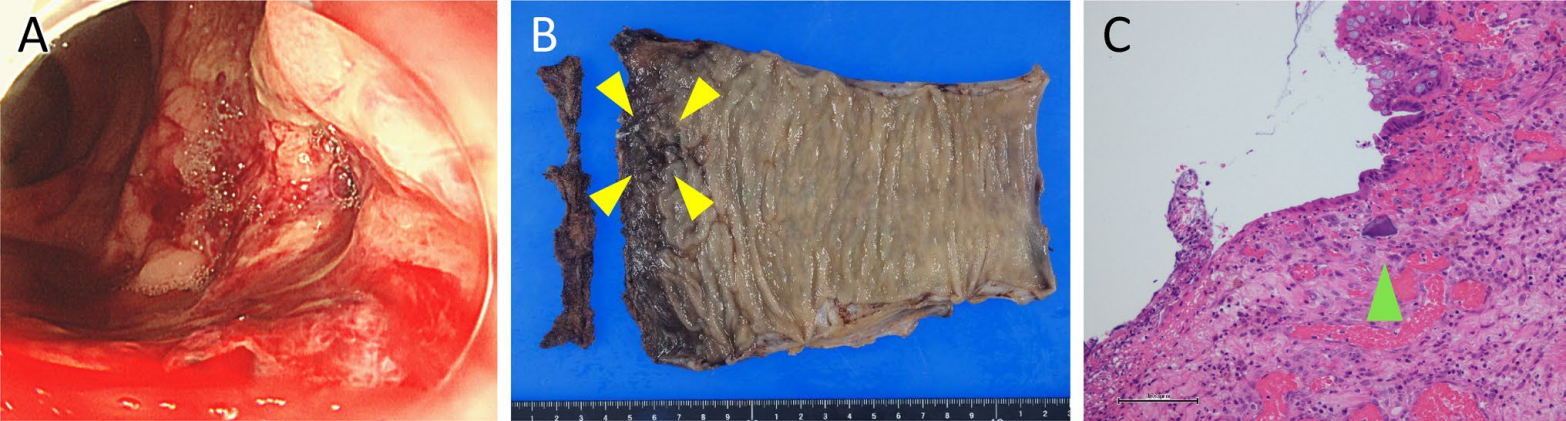
Endoscopic and pathologic findings of the rectal ulcer. **A** Colonoscopy showing rectal ulcer with hemorrhage. **B** The resected rectum showing a rectal ulcer near the dentate line (arrowheads). **C** Pathological findings showing basophilic crystalline material at the rectal ulcer (arrowheads)

On the 6th day, he suddenly developed hematochezia, and an emergent colonoscopy revealed an acute hemorrhagic rectal ulcer (AHRU) (Fig. 2A). On the 13th day, we performed transesophageal echocardiography and newly found a 20-mm vegetative structure on his damaged mitral valve (Fig. 1E), leading to a diagnosis of *E. coli*-associated IE. Bleeding from the AHRU was uncontrollable, even after repeated clipping by colonoscopy, and the patient underwent rectal resection and colostomy. Pathological findings of the resected colon showed deposition of crystalline material at the location of the rectal ulcer, corroborating the diagnosis of CPS-induced rectal ulcer (Fig. 2B, C). Mitral valve replacement surgery was performed immediately after rectal resection; however, the patient died the day after the operation.

## Discussion

This report describes a fatal case of *E. coli-*associated IE that was associated with CPS-induced rectal ulcers. This case highlights the potential risk of long-term use of CPS, resulting in colorectal ulcers, subsequent bloodstream infection, and consequently development of endocarditis.

*E. coli*-associated IE is a rare disease with high rates of complications, surgical interventions, and mortality that commonly involves elderly patients with risk factors such as diabetes mellitus or renal failure [3–5]. According to a literature review, 12% of reported patients with *E. coli*-associated IE were complicated with orthopedic lesions such as osteomyelitis and spondylodiscitis [3]. The source of infection is usually the urinary or biliary tract [3–8]. However, there were no underlying diseases at such frequent entry sites in the present case. On the other hand, the patient was found to have rectal ulcers that were pathologically confirmed to be induced by CPS, a cation-exchange resin. CPS is used for the treatment of hyperkalemia, but may occasionally cause hard stools and constipation as adverse effects. Recently, CPS has been reported to cause intestinal mucosal damage and colorectal ulcers [9–11]. CPS-induced rectal ulcers can be undermined by diabetes mellitus and renal failure, as observed in the present case [9]. The CPS-induced ulcer was reportedly diagnosed by the detection of crystal-like materials, along with dense infiltrations of lymphocytes, plasma cells, and neutrophils in the lamina propria [9–11]. In this case, histopathological examination revealed the presence of crystalline deposits and inflammatory cells at the ulcer base, leading to the diagnosis of a CPS-induced ulcer.

AHRU is a severe form of rectal ulcer, complicated by a sudden onset, painless, massive hematochezia from solitary or multiple rectal ulcers in elderly patients with atherosclerosis and diabetes mellitus [12]. AHRU typically develops in patients who are bedridden or wheelchair dependent. In this case, AHRU was considered to be precipitated by worsened general conditions due to preceding events of stroke and *E. coli* bacteremia. Although no prior endoscopic images were available, multiple ulcers in addition to the hemorrhagic site suggested the presence of stercoral ulcers. Rectal ulceration due to fecal impaction should be considered in geriatric patients exhibiting altered gastrointestinal motility, particularly chronic constipation [13, 14]. We assume that the CPS-induced rectal ulcer caused *E. coli* bacteremia, consequently leading to AHRU.

Collectively, the present case highlights the importance of evaluating colonic lesions in patients with *E. coli* bacteremia in the absence of underlying urinary or biliary tract infections. In addition, we should note that long-term use of CPS in patients with risk factors such as diabetes mellitus or renal failure may cause colorectal ulcers, possibly resulting in fatal bacteremia.

## Data Availability

The datasets used during the current study available from the corresponding author on reasonable request.
